# Value of contrast-enhanced ultrasound assisted core needle biopsy in the diagnosis of cervical lymph node tuberculosis

**DOI:** 10.3389/fonc.2025.1570133

**Published:** 2025-04-17

**Authors:** Jun Li, Dan Li, Wenzhi Zhang

**Affiliations:** Department of Ultrasonography, Hangzhou Red Cross Hospital (Integrated Chinese and Western Hospital of Zhejiang Province), Hangzhou, Zhejiang, China

**Keywords:** tuberculosis, contrast-enhanced ultrasound, core needle biopsy, cervical, lymph node

## Abstract

**Aim:**

To investigate the value of contrast-enhanced ultrasound (CEUS) assisted core needle biopsy (CNB) in the diagnosis of cervical lymph node tuberculosis (LN TB) and improve the positive rate of cervical LN TB.

**Methods:**

We retrospectively analyzed 730 samples obtained from July 2010 to January 2025 from patients treated with effective antituberculosis therapy and with microbiologically confirmed and surgical pathologically proven cervical lymph node enlargement who had undergone CEUS- CNB at our hospital. All patients were divided into two groups according to the historical control method. The CEUS group (2017–2025) underwent CEUS- CNB (485 cases), whereas the US group (2010–2018) underwent US-guided CNB (245 cases). The positive rates of pathological diagnosis and Xpert Mycobacterium tuberculosis complex (MTBC) and resistance to rifampin (RIF) (MTB/RIF) assay diagnoses were compared between the groups.

**Results:**

The specimens’ integrity was significantly higher after CNB in the CEUS group than in the US group (CEUS group: 72.30%; US group: 45.49%), and visual satisfaction of sampling in the CEUS group was higher (χ2: 47.651, P < 0.001). Histopathological examination sensitivity, specificity, positive predictive value, and negative predictive value were higher in the CEUS group than in the US group. The sensitivity of the Xpert MTB/RIF assay was significantly higher in the CEUS group than in the US group.

**Conclusion:**

The study results support the clinical use of CEUS for improving the diagnostic performance and positive rate for cervical LN TB.

## 1 Introduction

The World Health Organization’s 2024 Global Tuberculosis Report estimated 10.8 million new cases of tuberculosis (TB) worldwide in 2023 (1). In China, there were 748,000 new TB cases in 2022, with an incidence rate of 52,100,000, accounting for 7.1% of global TB cases and ranking third among countries with a high TB burden (2, 3). Lymph node tuberculosis (LN TB) is the most common type of extrapulmonary tuberculosis (EPTB) that usually occurs in the neck and is clinically manifested as enlarged cervical lymph nodes. LN TB accounts for approximately 30-35% of all EPTB cases (4, 5).

Early diagnosis and treatment directly affect the prognosis of cervical LN TB. Histopathological examination (HPE) of specimens after core needle biopsy (CNB) and Xpert Mycobacterium tuberculosis complex (MTBC) and resistance to rifampin (RIF) (MTB/RIF) assay can meet the needs of clinicians. CEUS-CNB of cervical lymph node diseases can improve the positive rate of pathological diagnosis (6). This study aimed to summarize and evaluate the value of CEUS- CNB in the diagnosis of cervical LN TB as well as evaluate its efficacy and safety.

## 2 Materials and methods

### 2.1 Study design

We included 730 patients with suspected cervical LN TB who were admitted to the Hangzhou Red Cross Hospital between July 2010 and January 2025. In these patients, cervical LN TB was later confirmed through biological culture and surgical pathological analysis. Subsequently, these patients were treated clinically with anti-TB therapy. The clinical manifestations in these patients included a neck mass, mild or no pain due to pressure, redness and swelling of the neck skin, and even sinus formation. After antibiotic treatment, the patients showed no effect or even worse symptoms. Among these patients, 99 were complicated with pulmonary TB, 21 with renal TB, and 78 with abdominal TB.

All patients were divided into two groups via the historical control method: CEUS group and US group. The CEUS group (485 cases, April 2017–January 2025) underwent CEUS- CNB, whereas the US group (245 cases, July 2010–September 2018), underwent US-guided CNB. All tissue samples were tested via HPE and Xpert MTB/RIF assay. The positive rates of HPE and Xpert MTB/RIF assay were compared between the two groups.

The inclusion criteria were as follows: (1) suspected cervical LN TB; (2) longitudinal LN diameter > 1 cm, transverse diameter > 0.5 cm, and an aspect ratio < 2; (3) no history of anti-TB treatment, no coagulation dysfunction, normal platelet range, no serious cardiovascular or cerebrovascular diseases; (4) no mental illness and could cooperate with completion of US-CNB surgery; (5) no severe allergies; (6) age > 18 years; and (7) a skin puncture area without damaged large area or ulcer.

Exclusion criteria: (1) patients under 18 years of age who could not undergo CEUS; (2) Lymph nodes are located near important blood vessels, or lymph nodes are surrounded by multiple blood vessels, and bleeding is inevitable; (3) Patients with abnormal coagulation function; (4) The skin at the puncture site is ulcerated in a large area, and the puncture point cannot avoid patients in the ulceration area.

In this study, CNB positive cases were those that tested positive in the HPE, Xpert MTB/RIF assay, or both. For patients with neck lymphatic periaccessory abscesses, we collected as many abscess samples as possible using syringes and performed MTB cultures and Xpert MTB/RIF assay to assist the diagnosis.

This study was approved by the Human Research Ethics Committee of Hangzhou Red Cross Hospital. All procedures were performed in accordance with the relevant guidelines and regulations or the Declaration of Helsinki. To ensure patient confidentiality and data privacy, all patient data were anonymized and stored in a secure, password-protected database accessible only to authorized personnel.

To determine the diagnostic accuracy of cervical LN TB in patients after CNB surgery, we selected a comprehensive reference standard (CRS) consisting of three components: effective anti-TB therapy, postoperative pathological diagnosis of cervical LN CNB specimens (pathological diagnosis of surgically resected specimens), and TB culture positivity. The diagnosis of LN TB in the neck was based on a positive HPE or Xpert MTB/RIF assay result after CNB or both (at least one positive result in HPE or Xpert MTB/RIF assay was required for a CNB positive diagnosis).

### 2.2 US and CEUS examination

We used a Philips ultrasonic diagnostic instrument (iU22, Philips Healthcare, Bothell) equipped with a high-frequency linear array probe (L12-5, frequency 5–12 MHz; L9-3, frequency 3–9 MHz) and a low-frequency convex array probe (C5-1, frequency 1–5 MHz) for imaging. All patients underwent routine bilateral cervical LN US examination. In the US group, LNs with abnormal structure, large volume on the affected side, and no large blood vessels and nearby important nerves were typically chosen as targets for CNB.

In the CEUS group, examination was performed using a low mechanical index (0.06) and pulse reverse harmonic imaging with a sulfur hexafluoride microbubble US contrast agent (SonoVue, Milan, Italy, Bracco SpA). Under electrocardiogram (ECG) monitoring, we injected 2.4 ml SonoVue into the elbow vein, followed by an injection of 5 ml of saline, close monitoring of the patient’s vital signs, and observation of the dynamic enhancement of the lesion for 2 min. All images were stored on the instrument’s hard drive for subsequent CEUS- CNB.

### 2.3 Diagnostic sample collection and processing

**Figure 1:**
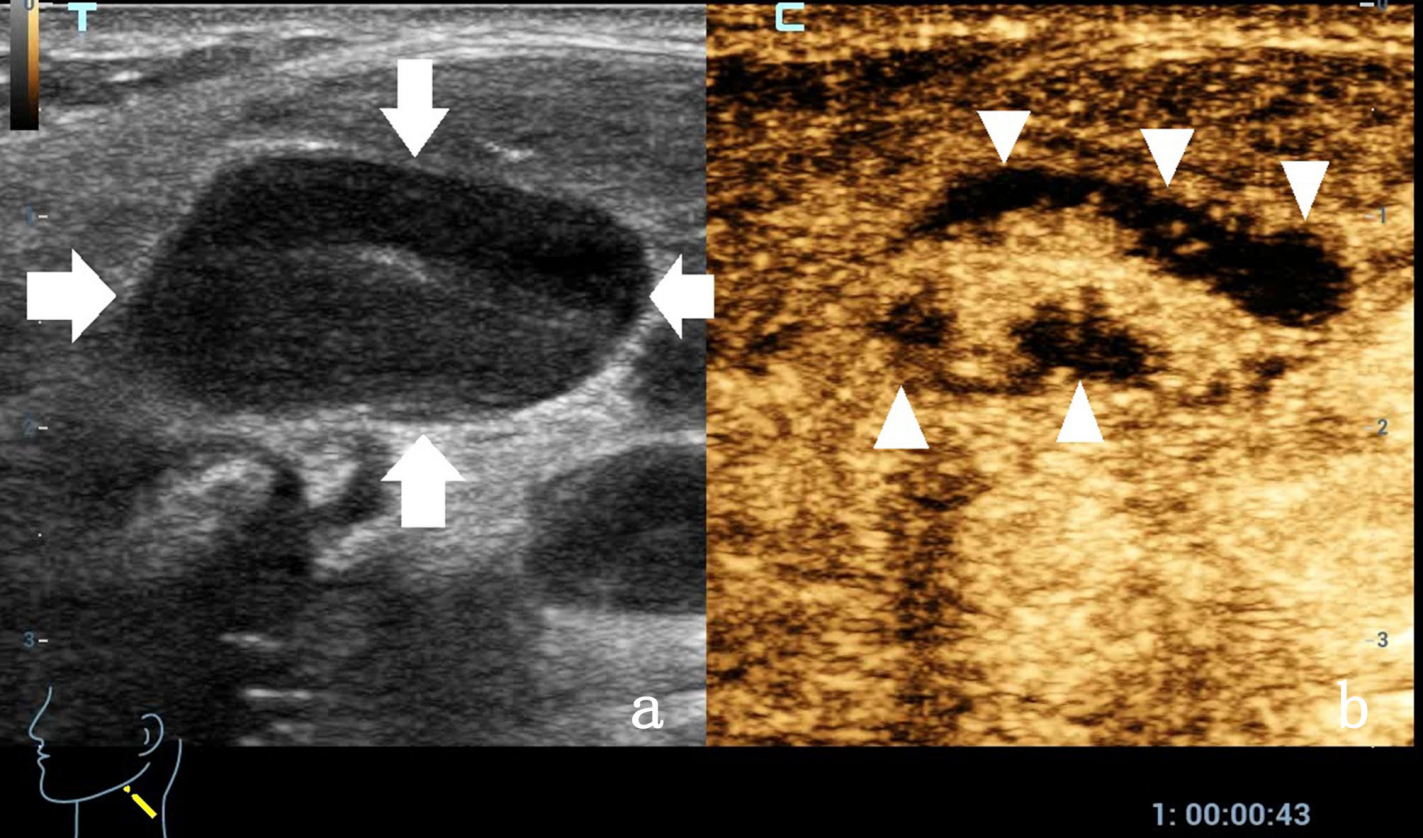
A 33-year-old man with enlarged lymph nodes in his left neck. **(a)** The conventional ultrasound control image shows enlarged lymph nodes with obvious cortical thickening and uneven internal echo. Arrows showing enlarged lymph nodes. **(b)** Contrast-enhanced ultrasound showing an irregular nonenhanced area (arrow head) in the lymph nodes, which was later confirmed as a necrotic area by core needle biopsy.

**Figure 2:**
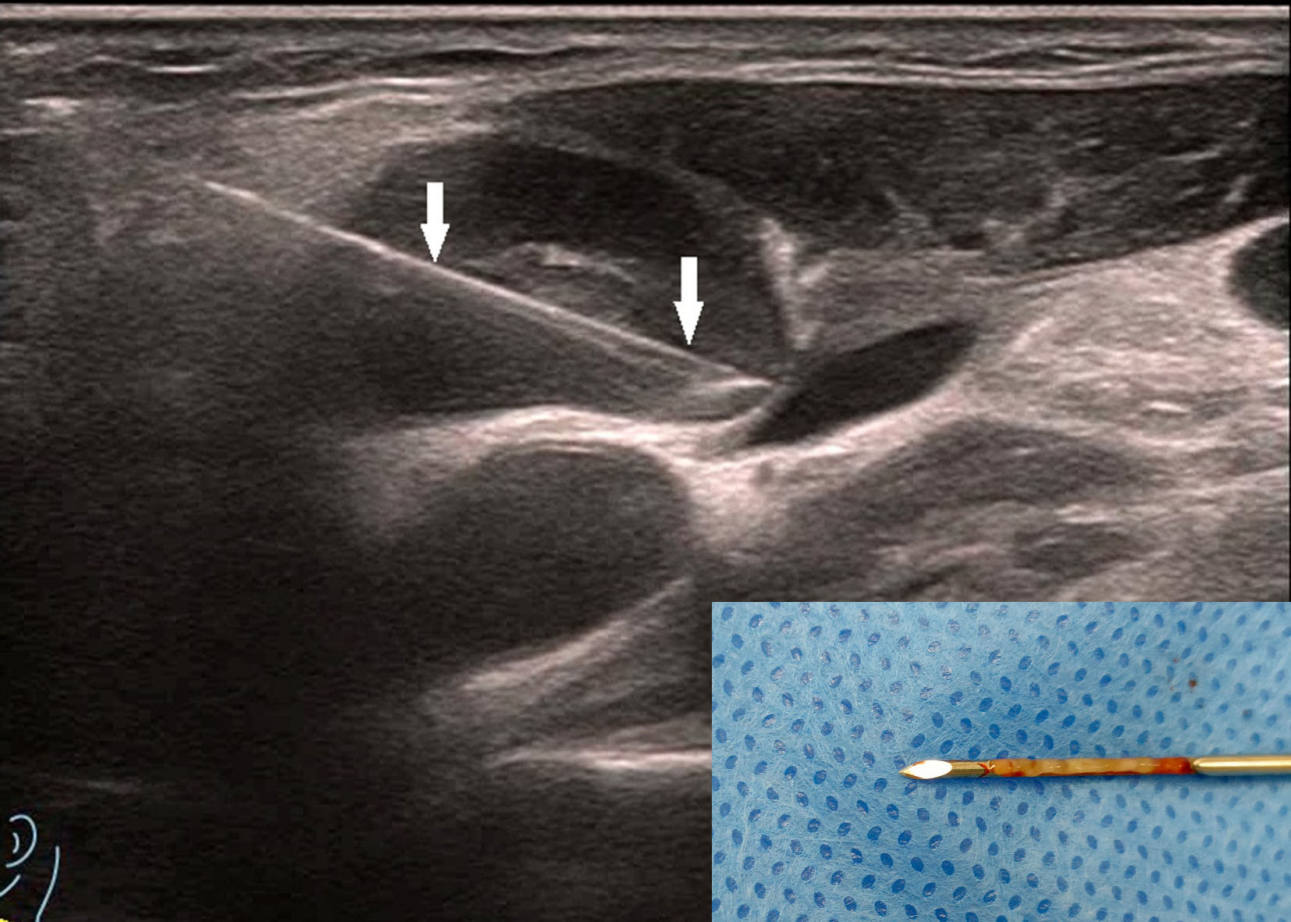
The patient is the same as shown in **Figure 1**. The target area for core needle biopsy (CNB) was the enhancement area, and the arrow shows the CNB needle. The figure on the right shows the good integrity of the puncture specimen. The patient was pathologically confirmed to have lymph node tuberculosis by Xpert MTB/RIF (+), The pathological results showed granulomatous inflammation with necrosis, suggesting tuberculosis.

**Figure 3:**
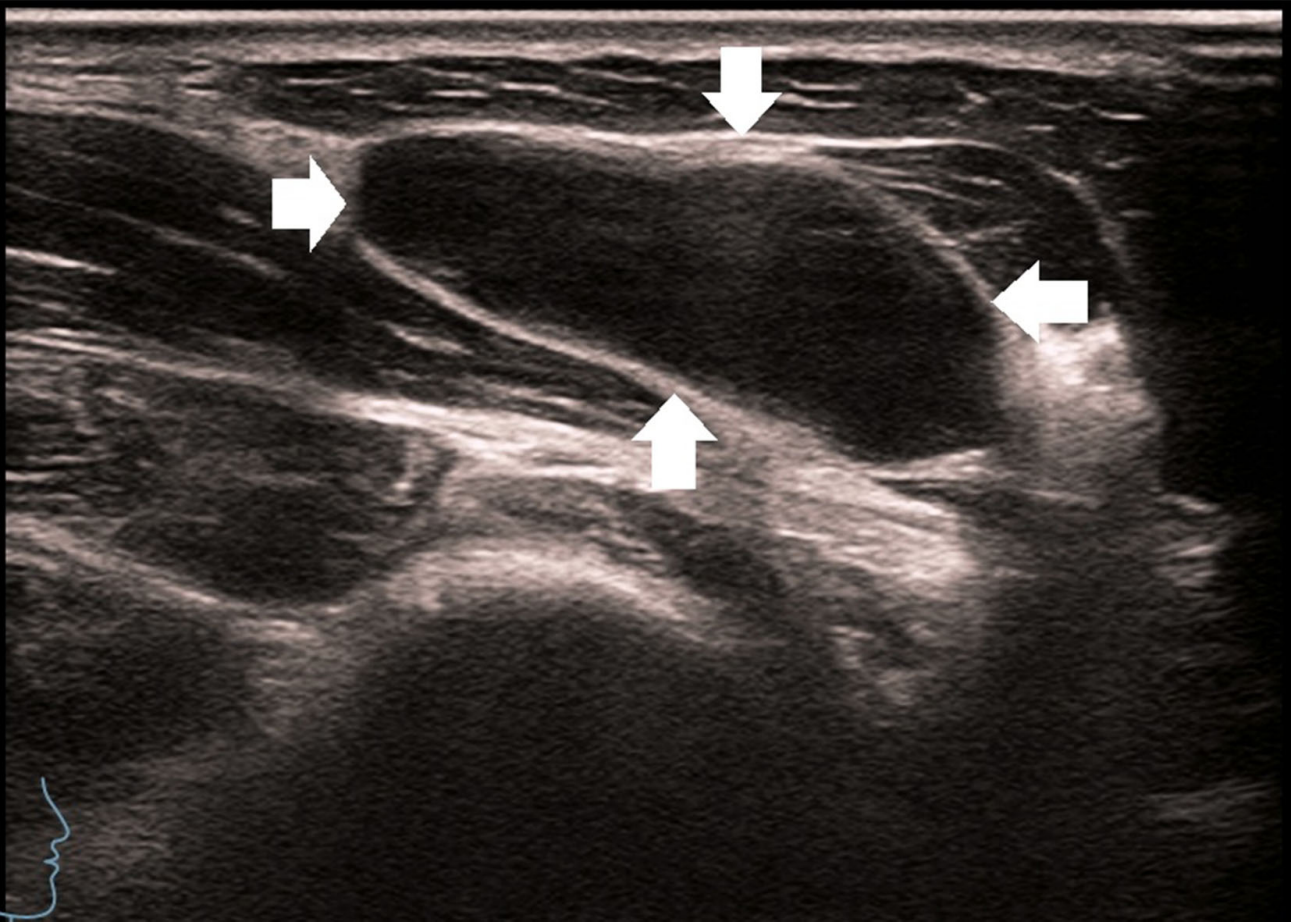
A 27-year-old man with enlarged lymph nodes in his right neck. Ultrasound showed significant enlargement of the lymph nodes, thickening of the cortex, disappearance of the lymphatic portal, and enlarged lymph nodes, as shown by the arrow.

**Figure 4:**
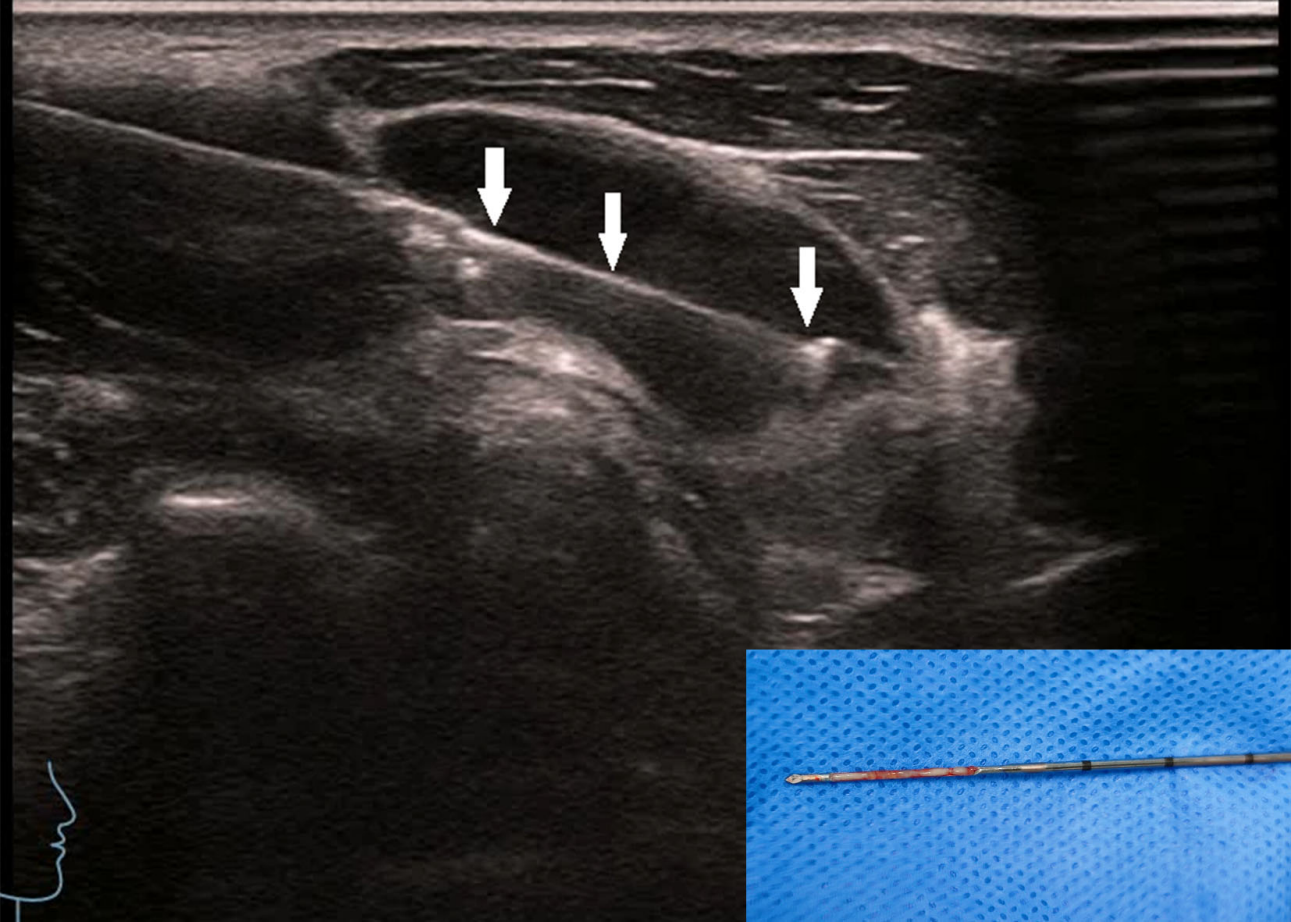
The patient is the same as shown in **Figure 3**. The biopsy was taken along the long axis of the lymph node. The arrow shows the core needle biopsy needle groove, and the image on the right shows good specimen integrity.

Before CNB, the patients were asked to lie in a supine or lateral position. The puncture site was disinfected under local anesthesia. A semiautomatic biopsy gun (18G, GALLINI S.R.L., ITALY) was used to obtain tissue samples. In the CEUS group, under the real-time guidance of US, a sampling area consisting of a fully enhanced area or mostly enhanced area plus a small amount of unenhanced area located in the enhanced area as much as possible was selected to improve the visual satisfaction of sampling and avoid biopsy of necrotic tissue (**Figures 1**, **2**). In the US group, under the real-time guidance of US, the longest sampling length of LN was used as the puncture path, and the sampling area was avoided as much as possible from the echoless area to improve the visual satisfaction of sampling (**Figures 3**, **4**). The sampling length of the two groups was approximately 1.0–2.0 cm, and the samples were taken twice from different directions. Two doctors with more than 5 years of experience in lymph node CNB independently analyzed the visual satisfaction of the samples, assessed the characteristics of specimen integrity, specimen discontinuity, and specimen paste, and recorded the results. Full tissue and discontinuous tissue in the core needle groove are regarded as satisfied with the specimen, and no tissue or liquid tissue is regarded as unsatisfied with the specimen. If the judgments of the two doctors were inconsistent, a third doctor with over 10 years of experience would assist in determining the final result. Pressure was applied for 15 min after CNB to prevent bleeding. Biopsy tissue specimens were usually divided into two parts: one for the HPE and one for the Xpert MTB/RIF assay. Tissue sample treatment reagents are added to the specimen and then ground until a suspension is formed. Place 2ml of the suspension into the Xpert MTB/RIF Reaction Kit (Cepheid, Sunnyvale, California). The reaction kit automatically detects TB. In this study, there were no serious complications such as massive bleeding, and 43 patients had mild pain, which disappeared one day after surgery.

### 2.4 Statistical analysis

We separately compared the diagnostic positive rates of cervical LN TB from the HPE and Xpert MTB/RIF tests between the two groups. SPSS ver. 23.0 statistical software (IBM Corp., Armonk, NY, USA) was used to analyze all data. The χ2 and Fisher’s exact tests were performed to analyze the numerical data and differences between the HPE and Xpert MTB/RIF assays. The inter-observer agreement was assessed using the Kappa statistic. A P value of <0.05 was considered statistically significant. The sample size was calculated based on a two-sample proportion comparison formula. Assuming a significance level (α) of 0.05, a statistical power (1-β) of 0.8, and an expected 10% higher diagnostic positive rate in the CEUS group compared to the US group, a minimum of 200 samples per group was required. To account for potential dropouts and incomplete data, we included 730 samples in total.

## 3 Results

### 3.1 Patients

In this study, 730 patients with suspected cervical LN TB were subjected to CEUS- CNB. The mean age of the patients was 33.35 ± 12.78 years (range: 19–70 years); 392 (53.69%) patients were 20–40 years old, 266 (36.43%) were 40–60 years old, and 72 (9.86%) were >60 years old. There were no significant differences in gender or age between the two groups. According to the CRS established in this study, 688 patients were diagnosed with cervical LN TB, 17 with metastatic cancer, 12 with necrotizing lymphadenitis, 9 with reactive hyperplasia, and 4 with nontuberculous mycobacterial infection. The most common feature of cervical LN TB was multiple enlargement of unilateral cervical LNs, followed by multiple enlargement of bilateral cervical LNs and single enlargement of unilateral cervical LNs (**Table 1**).

**Table 1:** Main clinical presentation of 730 patients with suspected of cervical LN TB.

Location	Number (n)
Left	249
Right	271
Bilateral	210
Main symptoms	
Cervical mass	311
Cervical tenderness	74
Skin redness and swelling	114
Cutaneous sinus	31
fever	378

### 3.2 US, CEUS, and CNB

The integrity and characteristics of the specimens of patients with cervical LN TB (688 cases) are shown in **Table 2**. In 42 patients with non-LN TB, the sample integrity was 88.09% (37 cases), with discontinuous tissue in 11.90% (5 cases) and mushy and liquid/non-tissue in 0% (0 cases). The inter-observer agreement between the two doctors for the assessment of specimen integrity, specimen discontinuity, and specimen paste was evaluated using the Kappa statistic, with Kappa values of 0.766, 0.789, and 0.897, respectively.

**Table 2:** Visual satisfaction of sampling of 688 patients with cervical LN TB.

	CEUS (455)	US (233)	χ^2^	*P*
Specimen integrity	329 (72.30%)	106 (45.49%)	47.651	<0.001
Specimen discontinuity	122 (26.81%)	89 (38.19%)	9.392	0.002
Specimen paste	4 (0.87%)	17 (7.29%)	21.443	<0.001
Liquid specimen/no tissue	0	21 (9.01%)	43.30	<0.001

In total, 688 patients with cervical LN TB were enrolled, including 479 Xpert MTB/RIF positive cases and 577 HPE positive cases. The sensitivity, specificity, positive predictive value (PPV), and negative predictive value (NPV) of Xpert MTB/RIF and HPE in the two groups are shown in **Tables 3**, **4**. Statistical analysis revealed that the CEUS group had higher HPE sensitivity, specificity, PPV, and NPV than the US group. More-over, the CEUS group had significantly higher Xpert MTB/RIF detection sensitivity than the US group.

**Table 3:** sensitivity, specificity, PPV and NPV of histopathology examination in two groups of patients with cervical LN TB.

Test	CRS		Sensitivity	Specificity	PPV	NPV	AUC
US	174	9	76.99% (70.95-82.31%)	52.63% (28.86-75.55%)	95.08% (90.87-97.73%)	16.13% (8.02-27.67%)	0.65 (0.58-0.71)
	52	10					
CEUS	403	4	88.96% (85.71-91.70%)	87.50% (71.01-96.49%)	99.02% (97.50-99.73%)	35.90% (25.34-47.56%)	0.88 (0.85-0.91)
	50	28					
*P*			<0.001	<0.01	<0.01	<0.01	

CRS, comprehensive reference standard; NPV, negative predictive value; PPV, positive predictive value; AUC, Area Under Curve.

**Table 4:** sensitivity, specificity, PPV and NPV of Xpert MTB/RIF assay in two groups of patients with cervical LN TB.

Test	CRS		Sensitivity	Specificity	PPV	NPV	AUC
US	145	0	65.61% (58.94-71.85%)	100.00% (73.54-100.00%)	100.00% (97.94-100.00%)	13.64% (7.25-22.61%)	0.83 (0.77-0.87)
	76	12					
CEUS	334	0	78.59% (74.38-82.40%)	100.00% (88.43-100.00%)	100.00% (98.90-100.00%)	24.79% (17.40-33.46%)	0.89 (0.86-0.92)
	91	30					
*P*			<0.001			0.047	

CRS, comprehensive reference standard; NPV, negative predictive value; PPV, positive predictive value; AUC, Area Under Curve.

The results of complications in perioperative patients with suspected cervical LN TB who had undergone CNB were as follows: median blood loss, 1 ml; operation time, 11 min, and hospital stay length, 2 days. No postoperative complications, such as severe bleeding, were observed.

## 4 Discussion

The gold standard for diagnosing cervical LN TB is biological culture and pathological diagnosis (7). The sensitivity of MTB culture for TB diagnosis using surgically excised tissues as specimens has been reported to be 18%–93% (8), but the surgery is more traumatic in the cervical area.

Fine-needle aspiration cytology (FNAC), which has low invasiveness and is easy to perform, has been used as a diagnostic method for cervical LN diseases (9). However, only a small number of specimens can be collected via FNAC, which directly affects the positive rate of diagnosis. The sensitivity of FNAC for the diagnosis of lymphadenopathy averages 90%, with a specificity of 95% (10). However, the sensitivity of FNAC for the diagnosis of infectious diseases is significantly low. FNAC has been used for the diagnosis of breast TB, and 25% of cases having cultured MTB were detected (11). FNA specimens were collected to examine cell morphological changes, growth of acid-fast bacillus, and cultured mycobacterium under a microscope. The sensitivity was 88.4%, and the specificity was 48.8% (12). Therefore, US-CNB was used in this study to diagnose cervical LN TB. US-CNB is currently the preferred method for obtaining a definitive histopathological diagnosis under nonsurgical conditions. The main pathological manifestations of cervical LN TB include exudation, caseous necrosis, and hyperplasia, accompanied by peripheral tissue edema of LNs, peripheral abscess, and sinus formation (13). The application of CEUS in the diagnosis of cervical LN diseases has increased (14, 15). CEUS can accurately reveal blood perfusion of LNs, thereby enabling the evaluation of the necrotic area within them. A previous study confirmed that obtaining diseased LN tissues was more effective with CEUS- CNB than with conventional CNB (16). Therefore, CEUS- CNB provides better evidence supporting the diagnosis of cervical LN TB.

EPTB is an oligobacterial disease, and the maximum incubation period of MTB culture is 8 weeks (17). Low bacterial content in specimens often leads to decreased sensitivity of MTB culture (18). The Xpert MTB/RIF assay can not only be used for the early diagnosis of cervical LN TB but also for determining the resistance of TB to rifampicin (19, 20), which is particularly important for patients with recurrent or drug-resistant cervical LN TB because it directly affects their prognosis, and the entire process can be completed within 2 h.

When tissue samples were used to diagnose TB, the HPE in this study appeared to be more effective than Xpert MTB/RIF for diagnosing cervical LN TB, which was consistent with the findings of a previous study (21). This finding may be related to the fact that our hospital is a provincial TB treatment center and the pathologists have extensive experience in TB diagnosis. It may also be related to the inclusion of the patient’s clinical symptoms and laboratory results in the pathology application form while submitting specimens for analysis, thereby influencing the pathologist’s diagnosis. The above finding may also be related to the fact that the selected specimens were mainly tissue. These factors need to be further studied by increasing the sample size and ideally should be confirmed based on the results of multicenter studies.

Among the patients with cervical LN TB in our study, the sample integrity after puncture biopsy was higher in the CEUS group than in the US group (CEUS group: 72.30%; US group: 45.49%); moreover, the visual satisfaction of sampling in the CEUS group was significantly higher, which was related to the fact that CEUS could accurately display the blood supply and determine the necrotic area in the lymph nodes, and the selection of samples could be made according to the CEUS results. Therefore, CEUS- CNB, referred to as traditional US-CNB, had a higher application value and was more scientific.

The HPE results showed that the HPE sensitivity, specificity, PPV, and NPV were higher in the CEUS group than in the US group. This finding may be related to the fact that after CEUS of cervical LNs, a surgeon can better grasp the sampling site, adjust and design the sampling direction, and obtain pathological tissue more effectively for analysis.

The Xpert MTB/RIF assay’s detection sensitivity was significantly higher in the CEUS group than in the US group; however, the specificity was the same, which may be related to the fact that the Xpert MTB/RIF assay is a molecular detection technology. On the one hand, the CEUS group had high satisfaction for sampling of gross samples and high sensitivity; on the other hand, the Xpert MTB/RIF assay had greater specificity for tuberculosis bacteria. Therefore, the detection specificity of LN TB was the same.

This study had some limitations. First, this was a retrospective single-center study, so the results need to be confirmed by performing a multicenter study. Second, the success rate of CEUS- CNB sampling somewhat depends on the experience of the surgeon. Finally, our hospital is a regional referral center with a medical policy that allows ad-mission of many patients with suspected LN TB from other hospitals. Our findings have important implications for further treatment.

## 5 Conclusion

In the diagnosis of cervical LN TB, CEUS- CNB showed higher postoperative specimen integrity and higher visual satisfaction of sampling than US-guided CNB. Moreover, CEUS- CNB had a higher pathological diagnosis positive rate than conventional CNB, and a higher diagnostic positive rate of Xpert MTB/RIF assay, making it a valuable tool for clinical applications. Another limitation of this study is that CEUS-CNB may lead to false positives and false positives, and this subset of patients needs to be prospectively studied and treated accordingly.

## Data Availability

The original contributions presented in the study are included in the article/supplementary material. Further inquiries can be directed to the corresponding author.

## References

[1] WHO. Global tuberculosis report 2024. Geneva, Switzerland: World Health Organization (2024).

[2] Kang W Yu J Du J Yang S Chen H Liu J. The epidemiology of extrapulmonary tuberculosis in China: A large-scale multi-center observation-al study. PloS One. (2020) 15:e0237753. doi: 10.1371/journal.pone.0237753 32822367 PMC7446809

[3] Kang W Liu S Du J Tang P Chen H Liu J. Epidemiology of concurrent extrapulmonary tuberculosis in inpatients with extrapulmonary tuberculosis lesions in China: a large-scale observational multi-center investigation. Int J Infect Dis. (2022) 115:79–85. doi: 10.1016/j.ijid.2021.11.019 34781005

[4] PHE. Tuberculosis in england 2019 report (presenting data to end of 2018). Public Heal Engl (2019).

[5] Cataño JC Robledo J. Tuberculous lymphadenitis and parotitis. Microbiol Spectr. (2016) 4(6). doi: 10.1128/microbiolspec.TNMI7-0008-2016 28084205

[6] Zhang WZ Yang GY Xu JP Zhang L Li J Zhao D. Comparative study of core needle biopsy and fine needle aspiration cytology in the diagnosis of neck lymph node diseases with contrast-enhanced ultrasound. Zhonghua Er Bi Yan Hou Tou Jing Wai Ke Za Zhi. (2016) 51:615–7. doi: 10.3760/cma.j.issn.1673-0860.2016.08.011 27625133

[7] MaChado D Couto I Viveiros M. Advances in the molecular diagnosis of tuberculosis: From probes to genomes. Infect Genet Evol. (2019) 72:93–112. doi: 10.1016/j.meegid.2018.11.021 30508687

[8] Fontanilla JM Barnes A von Reyn CF. Current diagnosis and management of peripheral tuberculous lymphadenitis. Clin Infect Dis. (2011) 53:555–62. doi: 10.1093/cid/cir454 21865192

[9] Adhikari P Sinha B Baskota D. Comparison of fine needle aspiration cytology and histopathology in diagnosing cervical lymphadenopathies. Australas Med J. (2011) 4:97–9. doi: 10.4066/AMJ.2011.559 PMC3562931 23386887

[10] Singh P Gupta N Dass A Handa U Singhal S. Fine needle aspiration cytology (FNAC) and neck swellings in the surgical outpatient. J Ayub Med Coll Abbottabad. (2008) 20:30–2. 19610510

[11] Tauro LF Martis JS George C Kamath A Lobo G Hegde BR. Tuberculous mastitis presenting as breast abscess. Oman Med J. (2011) 26:53–5. doi: 10.5001/omj.2011.14 PMC3191622 22043382

[12] Abdissa K Tadesse M Bezabih M Bekele A Apers L Rigouts L. Bacteriological methods as add on tests to fine-needle aspiration cytology in diagnosis of tuberculous lymphadenitis: Can they reduce the diagnostic dilemma? BMC Infect Dis. (2014) 14:720. doi: 10.1186/s12879-014-0720-z 25551280 PMC4299128

[13] Lekhbal A Chaker K Halily S Abada RL Rouadi S Roubal M. Treatment of cervical lymph node tuberculosis: When surgery should be performed? A retrospective cohort study. Ann Med Surg (Lond). (2020) 55:159–63. doi: 10.1016/j.amsu.2020.05.006 PMC7256428 32489658

[14] Zhang X Wang L Feng N Ni T Tang W. Reassessing the value of contrast-enhanced ultrasonography in differential diagnosis of cervical tuberculous lymphadenitis and lymph node metastasis of papillary thyroid carcinoma. Front Oncol. (2021) 11:694449. doi: 10.3389/fonc.2021.694449 34722243 PMC8551861

[15] Zhao D He N Shao YQ Yu XL Chu J Yang G. The diagnostic value of contrast-enhanced ultrasound for cervical tuberculous lymphadenitis. Clin Hemorheol Microcirc. (2022) 81:69–79. doi: 10.3233/CH-211355 35001882 PMC9108573

[16] Zhao D Shao YQ Hu J Liu D Tang W He N. Role of contrast-enhanced ultrasound guidance in core-needle biopsy for diagnosis of cervical tuberculous lymphadenitis. Clin Hemorheol Microcirc. (2021) 77:381–9. doi: 10.3233/CH-201038 33337357

[17] Wilkinson RJ Rohlwink UK Misra UK van Crevel R Mai NTH Dooley KE. Tuberculous meningitis. Nat Rev Neurol. (2017) 13:581–98. doi: 10.1038/nrneurol.2017.120 28884751

[18] Liu P Gu H Liu Y Qin Y. Application of core needle biopsy in the diagnosis of epididymal tuberculosis: a retrospective analysis of 41 cases. Int J Infect Dis. (2022) 122:33–7. doi: 10.1016/j.ijid.2022.05.044 35605951

[19] Steingart KR Schiller I Horne DJ Pai M Boehme CC Dendukuri N. Xpert^®^ MTB/RIF assay for pulmonary tuberculosis and rifampicin resistance in adults. Cochrane Database Syst Rev. (2014) 2014:CD009593. doi: 10.1002/14651858.CD009593.pub3 24448973 PMC4470349

[20] Boehme CC Nabeta P Hillemann D Nicol MP Shenai S Krapp F. Rapid molecular detection of tuberculosis and rifampin resistance. N Engl J Med. (2010) 363:1005–15. doi: 10.1056/NEJMoa0907847 PMC2947799 20825313

[21] Zhang W Xu J Zhang L Ni T. The value of histopathologic examination and Xpert (MTB/RIF) assay in diagnosis of cervical lymph node tuberculosis after coarse needle biopsy guided by CEUS: a retrospective analysis of 612 cases. Eur J Clin Microbiol Infect Dis. (2024) 43:1951–7. doi: 10.1007/s10096-024-04913-9 PMC11405491 39088108

